# In vivo imaging of systemic transport and elimination of xenobiotics and endogenous molecules in mice

**DOI:** 10.1007/s00204-016-1906-5

**Published:** 2016-12-20

**Authors:** Raymond Reif, Ahmed Ghallab, Lynette Beattie, Georgia Günther, Lars Kuepfer, Paul M. Kaye, Jan G. Hengstler

**Affiliations:** 1 Leibniz Research Centre for Working Environment and Human Factors, Technical University Dortmund, Dortmund, Germany; 2 Department of Forensic Medicine and Toxicology, Faculty of Veterinary Medicine, South Valley University, Qena, Egypt; 3 Centre for Immunology and Infection, University of York, York, UK; 4 Immunology and Infection Laboratory, QIMR Berghofer Medical Research Institute, Herston, QLD Australia; 5 Computational Systems Biology, Bayer Technology Services GmbH, Leverkusen, Germany

**Keywords:** Drug transport, Intravital imaging, Bile canaliculi, Hepatocyte, Lacteal, Glomerulus, Renal tubules

## Abstract

We describe a two-photon microscopy-based method to evaluate the in vivo systemic transport of compounds. This method comprises imaging of the intact liver, kidney and intestine, the main organs responsible for uptake and elimination of xenobiotics and endogenous molecules. The image quality of the acquired movies was sufficient to distinguish subcellular structures like organelles and vesicles. Quantification of the movement of fluorescent dextran and fluorescent cholic acid derivatives in different organs and their sub-compartments over time revealed significant dynamic differences. Calculated half-lives were similar in the capillaries of all investigated organs but differed in the specific sub-compartments, such as parenchymal cells and bile canaliculi of the liver, glomeruli, proximal and distal tubules of the kidney and lymph vessels (lacteals) of the small intestine. Moreover, tools to image immune cells, which can influence transport processes in inflamed tissues, are described. This powerful approach provides new possibilities for the analysis of compound transport in multiple organs and can support physiologically based pharmacokinetic modeling, in order to obtain more precise predictions at the whole body scale.

**Electronic supplementary material:**

The online version of this article (doi:10.1007/s00204-016-1906-5) contains supplementary material, which is available to authorized users.

## Introduction

**Fig. 1:**
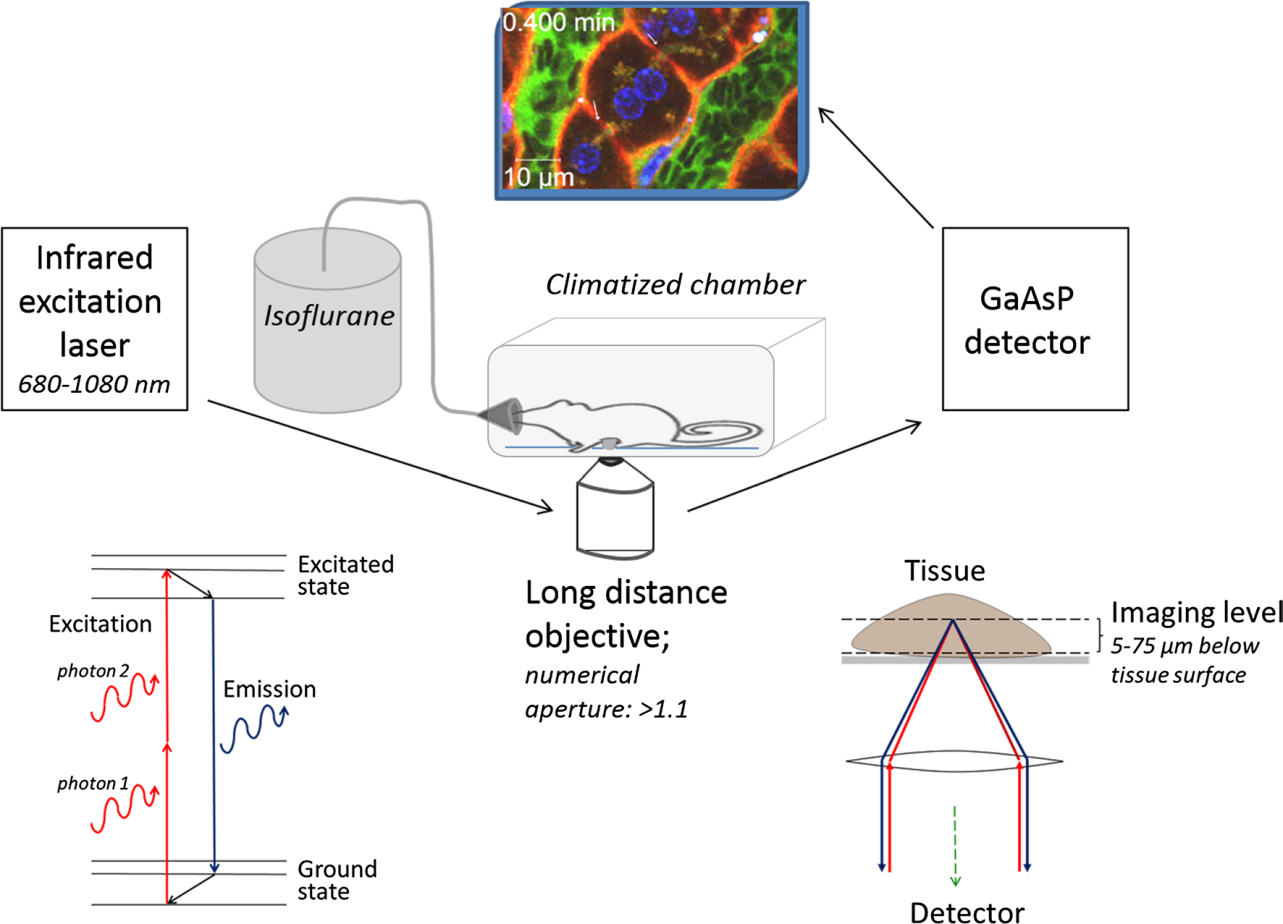
Setup for intravital imaging by two-photon microscopy. The infrared laser hits the fluorophore with two photons simultaneously each transferring half of the energy required for excitation. Excitation occurs only in the focal plane in the imaging level of the deeply anaesthetized mouse. Carefully prepared specimen, long-distance objectives with high numerical apertures and sensitive detectors ensure high-quality images of the investigated tissue

First-in-human trials represent a critical step in drug development due to interspecies differences between humans and the animal species in which preclinical research is performed. Recently it was shown that advanced techniques of physiologically based pharmacokinetic (PBPK) modeling improved the accuracy of extrapolation from mouse to human (Thiel et al. 2015). It has become clear that incorporation of species-specific physiology represents an important model parameter domain. However, even the most advanced PBPK models usually consider individual organs as single compartments. This may limit the accuracy of model simulations, since in reality, compound transport occurs in several sub-compartments of tissues which may show interspecies differences. Currently, only little is known about transport kinetics in sub-compartments within liver, kidney and intestine, because they have been difficult to analyze due to their structural complexity and the rapid kinetics of the processes involved. These difficulties may be overcome by the integration of data achieved via intravital two-photon imaging into PBPK models. A specific strength of two-photon microscopy, developed in the early nineteen nineties, is the possibility to image deeply into living tissues (Denk et al. 1990; Pittet and Weissleder 2011). This technique has been applied to study various biological processes in living organs, e.g., sterile tissue destruction by toxins (Marques et al. 2015a), pathogen infection (Beattie et al. 2013), tumor cell migration (Wolf et al. 2003), behavior of immune cells (Lammermann et al. 2013) as well as endocytosis (Masedunskas and Weigert 2008). Two-photon microscopy offers the possibility to study processes with fast kinetics that are impossible to analyze by conventional histology. Examples are transport processes of fluorescently labeled compounds, e.g., bile salts, proteins or xenobiotics that can be studied in a time-resolved quantitative manner. Moreover, the sequences of immune system associated events can be analyzed, for example tracing the death of parenchymal cells, which may be either the consequence or cause of immune cell infiltration (Inverso and Iannacone 2016; Marques et al. 2015a; Melgar-Lesmes and Edelman 2015). The resolution of approximately 200 nm, which can be achieved by two-photon microscopy, is appropriate for analysis of subcellular structures, such as mitochondria or vesicles. On the other hand, imaging at lower magnification gives an overview of the organization of larger tissue regions, such as several liver lobules or kidney nephrons. Recently, a state-of-the-art protocol on intravital real-time imaging of the liver has been published using conventional confocal microscopy (Marques et al. 2015b). However, this technique has two limitations: First, conventional confocal microscopy uses shorter wavelengths for excitation implying higher phototoxic potential. This may still be tolerable for slow frame rates. However, rapid processes such as trafficking or transport of molecules require faster sequences of recording; whereby, special care must be taken to avoid compromising cell function due to phototoxicity (Helmchen and Denk 2005; Zipfel et al. 2003). Second, penetration into living tissue by confocal microscopy is limited because of scattering and light absorption at the required laser wavelengths (Smith et al. 2009). Since excitation in two-photon-based imaging is achieved by two distinct photons, which meet at the focal plane (Fig. 1), energy transfer to the tissue is strongly reduced. Careful adjustment of recording facilitates fast imaging with minimal phototoxicity. In this context, it is of advantage that a single excitation wavelength can be used in two-photon imaging in order to emit several fluorophores throughout the spectrum. Therefore, acquisition is possible for several emission wavelengths simultaneously in a single track, which cannot be achieved with standard confocal microscopes.

In the present study, we introduce a toolbox for intravital two-photon-based imaging which can be applied for the major organs involved in compound uptake, metabolism and elimination including liver, kidney and intestine. Key features of the imaging setup are a two-photon laser with a broad wavelength spectrum, long-distance objectives with high numerical aperture, sensitive detectors like GaAsP, a precisely adjusted inhalation anesthesia and animal preparations by a trained expert (Fig. 1). A fundamental prerequisite to facilitate successful intravital imaging is appropriate fluorophores and reporter mice producing bright and stable fluorescence (Table 1). The mT/mG mouse offers great opportunities to visualize tissue morphology. This transgenic mouse line was generated by inserting a targeting vector encoding a floxed membrane-targeted tandem dimer tomato sequence, followed by a membrane-targeted enhanced GFP (eGFP) sequence, into the Gt(ROSA)26Sor locus (Muzumdar et al. 2007). In these mice, all cells express red fluorescence on their cell membranes. Moreover, by mating mT/mG mice with mice expressing Cre recombinase under control of tissue-specific promoters, a switch to membrane-targeted eGFP expression in the cells of interest can be achieved. For example, mice expressing Cre recombinase under control of the lysozyme M (LysM) promoter mated to mT/mG mice represent an attractive model to visualize macrophages and granulocytes. Similarly, Tie2Cre mice can be used to visualize eGFP-labeled endothelial cells. As an alternative to reporter mice, fluorophore-labeled antibodies against surface marker proteins allow imaging of specific cell types (Table 1). Particularly helpful are the cationic fluorescent dyes tetramethylrhodamine ethylester (TMRE) or rhodamine 123 which are used to study mitochondrial membrane potential. Since many cells are rich in mitochondria, TMRE or rhodamine 123 can also be injected to efficiently visualize tissue morphology.

**Table 1:** Selection of reporter mice, fluorescent marker dyes and antibodies for intravital imaging

	Indicated structure	Two-photon excitation range (nm)	Emission maximum (nm)	Concentration
Reporter mice				
mT/mG reporter	Tissue morphology	720–800	554	
Tie2 × mT/mG reporter	Tissue morphology differentiating endothelial cells	800–840	554/507	
LysM × mT/mG reporter	Tissue morphology differentiating macrophages and granulocytes	800–840	554/507	
Fluorescent marker dyes				
TMRE	Mitochondrial membrane potential	780–820	576	0.96 mg/kg
Rhodamine 123	Mitochondrial membrane potential	720–820	528	0.8 mg/kg
CLF	Bile acid transport	740–820	525	2.5 mg/kg
Fluorescein-labeled dextran (10 kDa)	Endocytic transport	740–820	525	30 mg/kg
Latex particles	Macrophages and endothelial cells	720–780	515	16 mg/kg
Alexa 546-labeled wheat germ agglutinin	Endothelial cells	780–800	573	1 mg/kg
Hoechst 33258	Nuclei	720–800	470	5 mg/kg
Conjugated antibodies				
PE-labeled CD11b	Infiltrating myeloid cells	720–780	575	60 µg/kg
PE-labeled NK1.1	NK cells and innate lymphoid cells	720–780	575	60 µg/kg
PE-labeled F4/80	Resident and infiltrating macrophages	720–780	575	60 µg/kg
PE-labeled Ly6G	Neutrophils	720–780	575	60 µg/kg

**Fig. 2:**
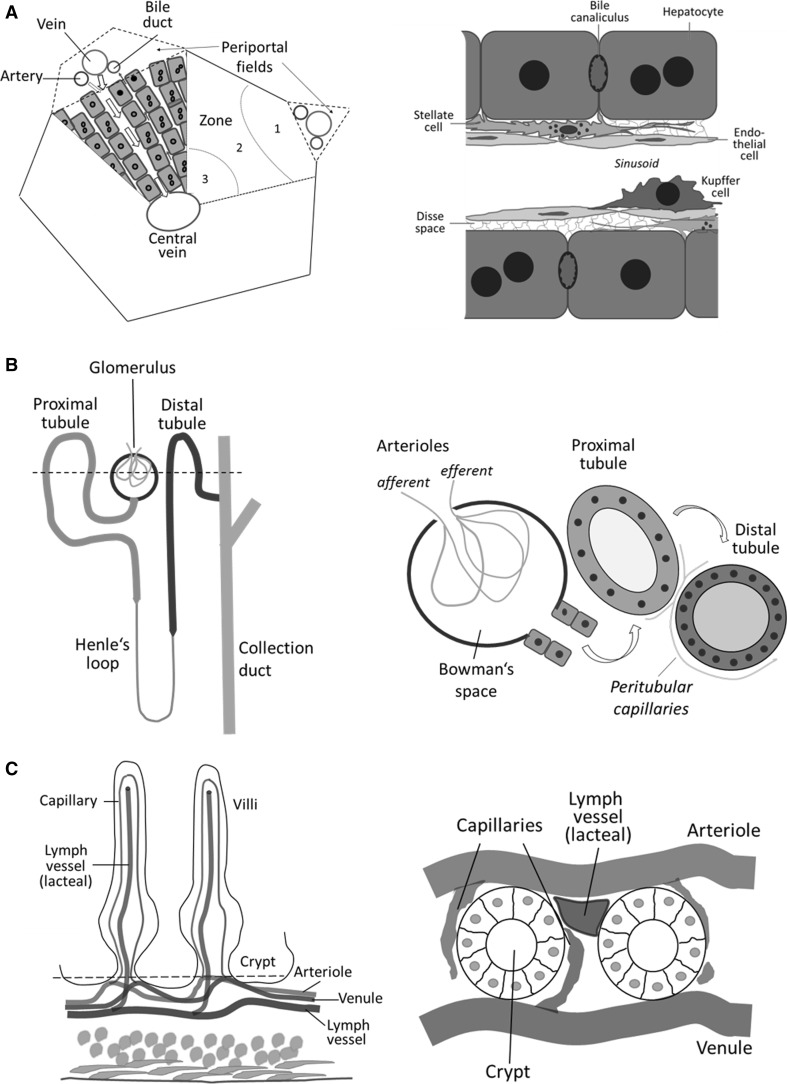
Schematic drawings of tissue structures found in intravital imaging. **a** Structure of the liver. *Left side* indicates details of the liver lobule. *Right side* depicts the cellular composition of the liver as seen in the videos generated by two-photon-based imaging. **b** Structure of the nephron. *Left side* indicates the approximate imaging level (*dashed line*) in the area of the convoluted tubules in a nephron. *Right side* depicts the morphological structures observed in intravital imaging including the Bowman’s space, and the proximal as well as distal tubules. **c** Structure of the duodenum. *Right side* indicates the approximate imaging level (*dashed line*) in the level of the crypt cells. *Right side* depicts observed morphological structures including blood vessels, capillaries and the lacteal

The presented methods can be applied to multiple organs, in the present study exemplified with liver, kidney and intestine. In the liver, hepatocytes, endothelial cells, Kupffer cells and infiltrating immune cells could be visualized (Fig. 2a). Moreover, the flux of fluorescent bile salts and drugs from the sinusoid via Dissé space and hepatocytes into bile canaliculi was quantified and the resolution of intravital imaging was sufficient to study vesicular transport. In the kidney, the intertubular blood vessels, the glomerular capillaries, the Bowman’s space, proximal as well as distal tubules and the collecting duct were imaged. Glomerular filtration into the Bowman’s space followed by tubular transport was quantified (Fig. 2b). The same approach allowed imaging of intestinal crypts and the transfer of drugs from capillaries to lymph vessels (lacteal) (Fig. 2c).

All recordings presented were taken from living, anaesthetized mice with intact organs exhibiting all biological processes in vivo. The videos presented represent raw data without any image processing like image registration or de-shaking. The quality of the recordings was sufficient for elementary quantifications by defining regions of interests in order to achieve kinetic data of the transport processes. The technique we describe facilitates in vivo recording to study systemic transport and excretion processes in the most important organs.

## Materials and methods

### Microscope setup

In vivo recordings were acquired using a custom modified inverted LSM MP7 (Zeiss, Jena, Germany) provided with a two-photon Chameleon Ultra II laser with a tuning range from 680 to 1080 nm and a maximum power of approximately 3.5 W at 800 nm. For detection in channel 1, a non-descanned detector with a short-pass filter at 485 was used. Channels 2 and 3 were equipped with a gallium arsenide phosphide (GaAsP) detector with a band-pass filter at 500–550 and 565–610, respectively. Images were acquired with an LD C-Apochromat 40×/1.1 water immersion objective. The temperature of the specimen was controlled with a matt coated incubation chamber (Solent Scientific, Segensworth, UK).

### Surgical procedure

For in vivo imaging, 6- to 10-week-old male mice were initially anaesthetized with an intraperitoneal injection of ketamine (64 mg/kg), xylazine (7.2 mg/kg), acepromazine (1.7 mg/kg) and buprenorphine (0.08 mg/kg). When mice did not show reflexes after pinching feet, the abdomen was shaved and covered with olive oil to avoid disturbance by cut hair in the field of imaging. For liver preparation, the abdominal skin was removed and a small midline incision below the sternum was made with surgical scissors. Subsequently, the falciform ligament was cautiously cut in order to dissociate the liver. The left liver lobe was carefully exposed by gently pushing the abdomen, and subsequently, the mouse was placed in a lateral position onto a 49 mm × 74 mm cover slip 0.17–0.16 mm thickness (Ocon 159, Logitech, Glasgow, UK). The cover slip was put in a custom-made image platform from Plexiglas and fit into the stage of the microscope. The exposed liver was then covered with saline-soaked gauze. The temperature control of the incubation chamber was set at 36 °C. The quality of mouse preparation was verified by checking the breathing rate of the mouse and by the regular blood flow in the observed region. For the preparation of the kidney, the mouse was placed on its right side and a small incision made in the left flank region. The convex side of the left kidney was exposed via gentle pressure on the abdominal wall. In order to avoid dryness, the exposed kidney was covered with saline-moistened gauze during imaging. For intestine imaging, an incision was made on the right side of the abdominal wall. The intestine was gently pulled out using a moistened cotton-tipped applicator. To reduce peristalsis, the intestine was incubated in a 1% atropine sulfate bath for 10 min. Subsequently, the intestine was placed in a glass bottom microwell dishes (MatTek corporation, MA, USA) containing 0.5% atropine sulfate bath during the entire recording period. In case of recordings exceeding 1 h, the mice were kept on maintenance anesthesia via a respiratory mask using an evaporator (IAS3801, FMI GmbH, Seeheim-Jugenheim, Germany) with 1–2% isoflurane and a subcutaneous injection of 0.5 ml saline was given.

### Reporters and markers

Wild-type mice used for this study were C57CL/B6N (Janvier Labs, Le Genest-Saint-Isle, France). mT/mG mice (Muzumdar et al. 2007) (#007676), Tie2Cre mice (Koni et al. 2001) (#004128) and LysMCre mice (Clausen et al. 1999) (#004781), all on the B6 background, were all obtained from the Jackson Laboratories, CA, USA. Tie2Cre and LysMCre mice were crossed with mT/mG mice to create cell-specific eGFP reporter mice.

Fluorescent markers or fluorescently labeled antibodies were injected via the tail vein before surgery or during recording via lateral tail vein catheters (SAI-infusion, IL, USA). Fluorescent marker dyes were TMRE and rhodamine 123, which had been solved in 50% methanol/PBS at a concentration of 1.2 mg/ml and 1 mg/ml, respectively (Thermo Scientific, MA, USA), cholyl-lysyl-fluorescein (CLF) in PBS at a concentration of 1 mg/ml (BD Biosciences, California, USA), fluorescein-labeled dextran 10,000 MW in PBS at a concentration of 10 mg/ml (Thermo Scientific), green fluorescent latex particles at a concentration of 50 mg/ml (Thermo scientific), Alexa 546-conjugated wheat germ agglutinin in PBS at a concentration of 0.5 mg/ml (Thermo Scientific) and Hoechst 33342 in PBS at a concentration of 10 mg/ml (Thermo Scientific). All injected antibodies including anti-mouse CD11b, anti-mouse F4/80, anti-mouse NK1.1 and anti-mouse Ly6G (eBioscience, CA, USA) were phycoerythrin conjugated and administered in 100 µl PBS.

### Animal models

For induction of acute liver damage, mice were injected intraperitoneally with 300 mg/kg APAP (Sigma-Aldrich, Taufkirchen, Germany) after overnight fasting. Three days after injection, livers of the mice were prepared for intravital imaging. In order to induce liver fibrosis, mice were injected intraperitoneally twice a week with 1 g/kg CCl_4_ (Carl Roth, Karlsruhe, Germany) for 2 months. Four days after, the last injection mice were prepared for intravital imaging.

### Quantification of compound uptake

Recorded videos were quantified with the Zen software (Zeiss). Fluorescence intensity was quantified in defined regions of interest.

### Pharmacokinetic analyses

CLF and dextran half-lives in the quantified fluorescence intensities were determined by linear regression with MATLAB (version 8.5.0.197613; The MathWorks, Inc., Natick, MA). Pharmacokinetic analyses were performed with the MoBi^®^ Toolbox for MATLAB (version 6.0.3; Bayer Technology Services GmbH, Leverkusen, Germany). PK-Sim^®^ and MoBi^®^ are freely available for non-commercial academic use. To this end, the quantified fluorescence intensities of CLF and dextran were treated as pharmacokinetic plasma concentration profiles and analyzed accordingly for their pharmacokinetic properties.

## Results

### Liver: from sinusoids to bile canaliculi

The functional unit of the liver is the lobule, which displays a specific architecture consisting of the parenchymal hepatocytes, the endothelial cells lining the sinusoids, the stellate cells and the resident macrophages called Kupffer cells (Fig. 2a).

#### Hepatocytes

**Fig. 3:**
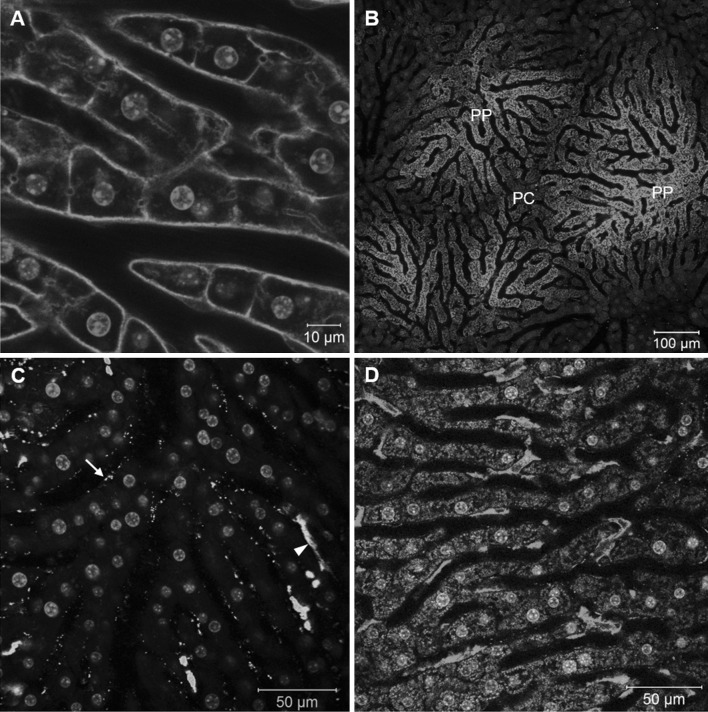
Intravital imaging of the liver. **a** Visualizing hepatocytes membranes by mT/mG mice. **b** TMRE visualizes lobular zonation with higher fluorescence in the periportal (PP) compared to the pericentral (PC) region. **c** *Green* fluorescent latex particles of 100 nm diameter are taken up by LSEC (*arrows*) and Kupffer cells (*arrowhead*). **d** Imaging of Kupffer cells (*red*) using fluorophore labeled F4/80 antibodies (color figure online)

The parenchymal cells, which are arranged in sheets along the sinusoids, can be easily identified in vivo using mT/mG mice (Fig. 3a). The resolution of the two-photon microscope is sufficient to detect single transport vesicles, with a particularly high vesicular density in the proximity of bile canaliculi (Video 1A, B). Due to their pronounced energy metabolism, hepatocytes can easily be visualized by mitochondrial membrane potential markers like TMRE or rhodamine 123, enabling differentiation of individual mitochondria within hepatocytes (Video 2). This video additionally shows a Kupffer cell, which can easily be differentiated from hepatocytes by their location in the sinusoidal lumen and the different mitochondrial morphology. Examination of the liver following TMRE and rhodamine 123 administration illustrates that mitochondrial markers can be applied to visualize lobular zonation (Fig. 3b). The mitochondrial staining marker appears bright in the periportal (PP) region and decreases gradually toward the lobular center (PC) due to the porto-central direction of the blood flow, which allows more uptake in the PP region.

#### Sinusoidal endothelial cells

Specific visualization of sinusoidal endothelial cells (LSECs) in fluorescence microscopy can be achieved with Tie2 × mT/mG reporter mice. These mice express membrane-targeted eGFP in the LSEC, while membranes of the other cell types of the liver remain red (Video 3). The LSECs line the sinusoidal lumen and their nuclei show only a weak Hoechst signal compared to hepatocytes. Remarkably, in Tie2 × mT/mG reporter mice further cells in the sinusoidal lumen also express eGFP. Among them, platelets appear as small flakes rapidly flowing through the sinusoids, which is helpful for identification of the direction of the blood flow. These platelets can form transient aggregates (Video 3, 21–34 min). LSEC can also be stained by fluorescently labeled latex nanoparticles. Particles with a diameter larger than 500 nm are known to be exclusively taken up by macrophages. In contrast, smaller sized particles, below 100 nm, are taken up by macrophages as well as by LSEC (Fig. 3c). While fluorescent latex nanoparticles visualize both LSEC and Kupffer cells, lectins such as wheat germ agglutinin preferentially bind to glycoproteins of LSEC (Video 6). Injection of fluorescently labeled lectins is therefore an additional method that can be used to mark the sinusoidal network.

#### Kupffer cells

In order to investigate the resident macrophages in the liver by intravital imaging, LysM × mT/mG reporter mice can be used (Video 4). These mice also allow the visualization of other immune cell types that express LysM, including monocytes and granulocytes. While the green fluorescent Kupffer cells remain stationary, their protrusions show highly dynamic micromotility. Macrophages of the liver can also be visualized by intravenous injection of fluorescently labeled marker antibodies such as F4/80 (Fig. 3d).

### Functional imaging of hepatic transport

**Fig. 4:**
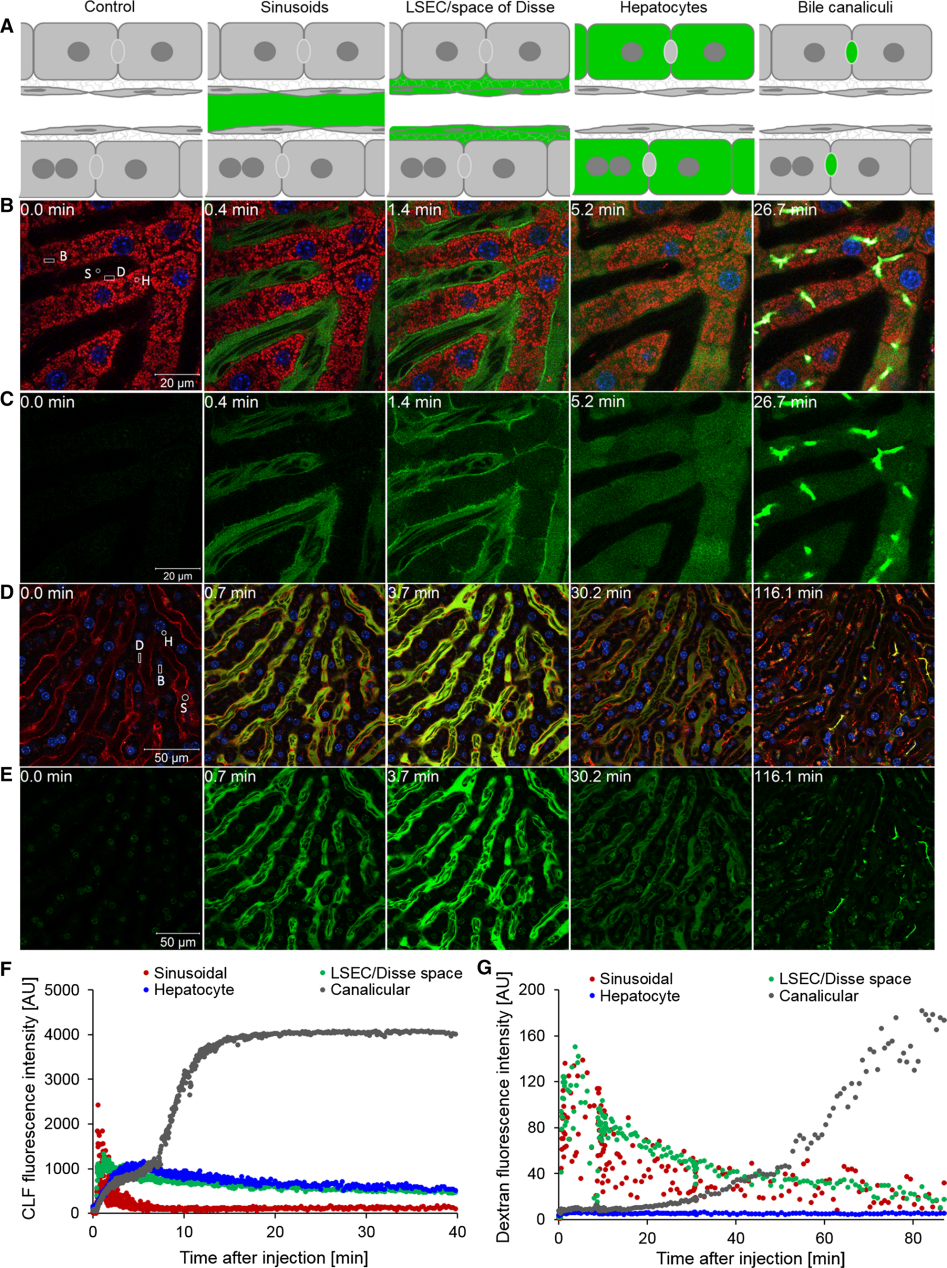
Hepatic transport of CLF and dextran. **a** Illustration of the four stages of during hepatic transport, namely appearance in the sinusoids, enrichment in the LSEC/Dissé space, uptake by the hepatocytes and transport into the bile canaliculi. **b** Stills representing the four stages after CLF injection from Video 5. Hepatic morphology was visualized by injection of TMRE (*red*) and transport of CLF was followed (*green*). **c** CLF channel only. **d** Stills representing the four stages after fluorescent dextran 10,000 MW injection from Video 6. Hepatic morphology is evident by the expression of membrane-targeted tomato (*red*) and transport of dextran was followed (*green*). **e** Dextran channel only. Quantification of the CLF (**f**) and dextran (**g**) kinetics in the sinusoidal blood (*S*), LSEC/Dissé space (*D*), hepatocytes (*H*) and bile canaliculi (*BC*). Positions of quantification are indicated by *white circles* and *rectangles* in the *left panels* of **b** and **d** (color figure online)

A frequently used method to study hepatic transport is via tracing of the fluorescently labeled bile salt, CLF (de Waart et al. 2010; Milkiewicz et al. 2000). After intravenous injection of 2.5 mg/kg CLF, the following states could be differentiated (Fig. 4a–c; Video 5): (1) CLF appearance in the sinusoids approximately 15 s after tail vein injection, followed by a rapid decrease in the sinusoids indicating an efficient systemic clearance, (2) transient enrichment of CLF in the LSEC/space of Dissé, (3) after approximately after 5 min a maximum homogenous signal in the cytosol of the hepatocytes was detected and followed by (4) rapid export into the bile canaliculi. CLF accumulated in the bile canaliculi to the highest local fluorescent intensity, which exceeded the peak in the hepatocyte cytosol (Fig. 4f).

Application of fluorescently labeled dextran with a molecular weight of 10 kDa showed different transport kinetics due to the transporter independent endocytic uptake (Fig. 4d, e). Dextran appeared in the liver sinusoids 15 s after tail vein injection; whereby, no enrichment in the LSEC/space of Dissé was apparent. Further, there was no apparent signal in the hepatocyte cytosol, but distinct vesicular structures were evident. Approximately 15 min after injection, dextran was detectable in the bile canaliculi and enriched progressively during the time of recording (Video 6A, B). The half-life of dextran in circulation was markedly longer (15.9 min) compared to CLF (1.9 min) and was therefore present in the sinusoids for the entire time of recording of almost 90 min (Table 2). The plots of the sinusoidal dextran signal depict a scattered pattern due to the lower level of fluorescence in erythrocytes which continuously interrupted the fluorescent signal in the blood (Fig. 4g).

**Table 2:** Calculated half-lives for CLF and dextran in different compartments of the liver, kidney and intestine

Organ	Compartment	Half-life for CLF (min)	Half-life for dextran (min)
Liver	Sinusoids	1.89	15.91
	LSEC/Dissé space	13.46	19.15
	Hepatocytes	34.17	nd^a^
	Bile canaliculi	nd^b^	nd^c^
Kidney	Glomerular capillaries	2.33	17.54
	Bowman’s space	2.19	6.57
	Proximal tubule	4.88	10.62
	Distal tubule	nd^d^	nd^e^
Intestine	Blood vessels	1.45	14.32
	Lymph vessels	nd^f^	35.01

nd: not detectable due to transportation in vesicles^a^ or no detected decrease in fluorescence within the recording time of 105 min^b^, 117 min^c^, 4 min^d^, 110 min^e^ and 4 min^f^

Intravenous injection of dextran, clinically used as plasma volume expander, showed uptake into hepatocytes and vesicular transport into bile canaliculi. Although excretion of dextran via bile has been reported (Pupyshev and Maiborodina 2002), direct observation of its vesicular transport across hepatocytes has not yet been possible. An as yet unknown feature of bile salt transport is its transient enrichment in the LSEC/Space of Dissé before CLF signal appears in the hepatocytes (Fig. 4b, middle). This might be due to bile salt adsorption to extracellular matrix components in the Dissé space, prior to active transport through the hepatocyte membrane. Compound enrichment in close proximity to the carriers of the basolateral hepatocyte membranes may improve uptake into hepatocytes. Unfortunately, the resolution of classical two-photon imaging is not sufficient to differentiate the Dissé space from the very thin endothelial cells. Therefore, it remains on known whether transient CLF accumulation occurs in the endothelial cells or in the Dissé space.

### Kidney: from glomeruli to distal tubules

**Fig. 5:**
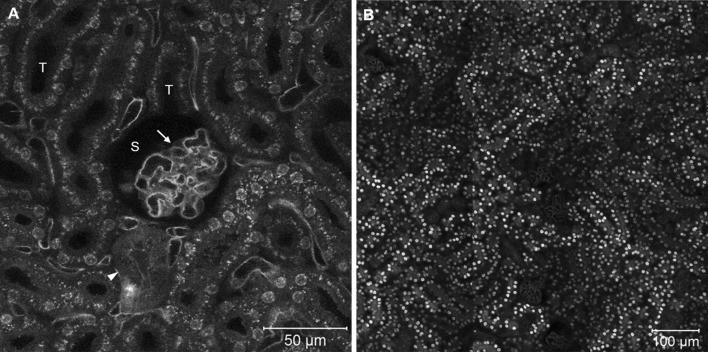
Intravital imaging of the kidney. **a** Endothelial cells of glomerular and intertubular capillaries appear *green* in Tie2 reporter mice. Further structures including glomeruli (*arrows*), Bowman’s space (*S*) tubules (*T*) and collecting ducts (*arrowheads*) are apparent. **b** Kidney morphology in a mT/mG mouse. *Images* were taken at the level of the convoluted part of the tubules as illustrated by the *dashed line* in Fig. 1 (color figure online)

Utilizing the same approach introduced for the liver, the functional unit of the kidney, the nephron, can also be visualized in vivo. mT/mG and Tie2 × mT/mG reporter mice display all major structural elements of the nephron including glomeruli, Bowman’s space, proximal and distal convoluted tubules and collecting ducts (Fig. 5). With the aid of Tie2 × mT/mG reporter mice, the endothelial cells of renal microvessels including glomerular capillaries as well as intertubular capillaries can be visualized (Fig. 5a). At the luminal side of the tubular cells, a high density of vesicles was observed, which mediate the tubular reabsorption of filtered substances. Cell membranes of the proximal and distal tubular epithelial cells are difficult to distinguish by light microscopy. TMRE as well as rhodamine 123 allowed the visualization of tubular cells (Videos 9, 10).

### Functional imaging of glomerular filtration and tubular transport

**Fig. 6:**
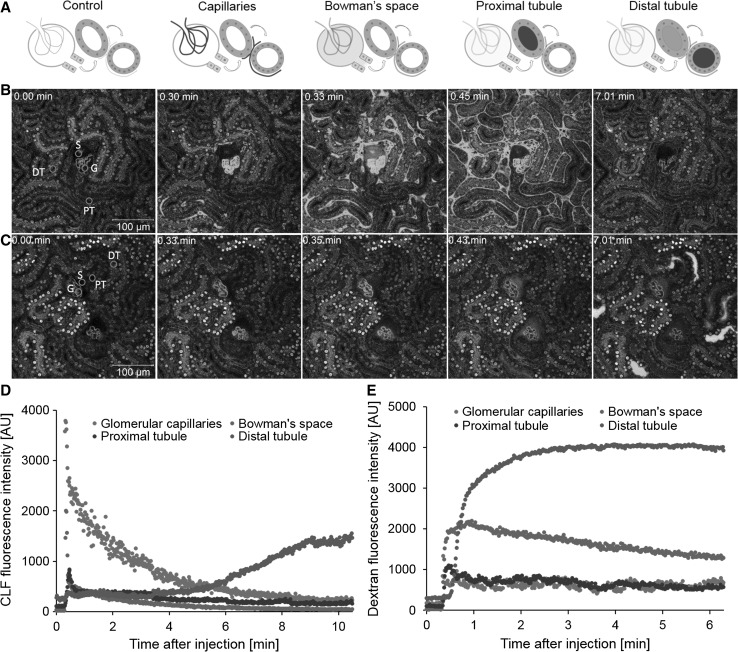
Renal transport of CLF and dextran. **a** Illustration of four stages of renal filtration and tubular transport: appearance in the glomerular and intertubular capillaries, filtration into Bowman’s space, transport to the proximal and distal convoluted tubules. Stills representing the four stages after CLF (**b**) or fluorescent dextran 10,000 MW (**c**) injection. *Images* represent stills from Videos 7 and 8. Quantification of CLF (**d**) and dextran (**e**) in the glomerular capillaries (*G*), Bowman’s space (*S*), proximal (*PT*) and distal convoluted tubules (*DT*). Positions of quantification are indicated by *green circles* in the *left panels* of **b** and **c**

In order to visualize systemic clearance by the kidney, a similar experimental setup to that used in the liver with a tail vein injection of CLF or dextran was chosen. In vivo recordings after CLF injection lead to differentiation of the following stages: (1) appearance of CLF in the intertubular and glomerular capillaries, (2) filtration into the Bowman’s space, (3) entrance into the proximal convoluted tubule and (4) entrance into the distal convoluted tubule (Fig. 6a, b; Video 7). Moreover, retention of CLF by the microvilli of the brush border cells of the proximal tubule was evident. CLF accumulation occurred also at the luminal side of distal tubule epithelial cells.

Quantification of the CLF signal showed rapid filtration from glomerular capillaries into Bowman’s space (Fig. 6d); whereby, the CLF in circulation is efficiently eliminated with a half-life of 2.3 min (Table 2). After filtration into Bowman’s space, CLF appeared in the proximal and later in the distal tubules. In the tubular lumen, a distinct retention by the brush border was evident, indicating a subsequent reabsorption mechanism for bile acids. Approximately 9 min after injection, CLF in the distal tubules was concentrated by a factor of two compared to the highest fluorescence intensity in the proximal tubules.

Due to its molecular properties, the renal elimination of fluorescently labeled dextran revealed significant differences compared to CLF. The filtration into Bowman’s space was less efficient due to its molecular size, with a threefold higher half-life (Fig. 6c; Video 8). After passing the Bowman’s space, dextran entered the proximal and the distal tubules showing significantly less retention in the brush border of the tubular cells (Fig. 6c). A striking feature was the long persistence of dextran in the distal tubules where no half-lives could be calculated for the time of imaging. The fluorescence intensity of dextran increased in the distal tubules approximately sixfold compared to the proximal tubules (Fig. 6e).

**Fig. 7:**
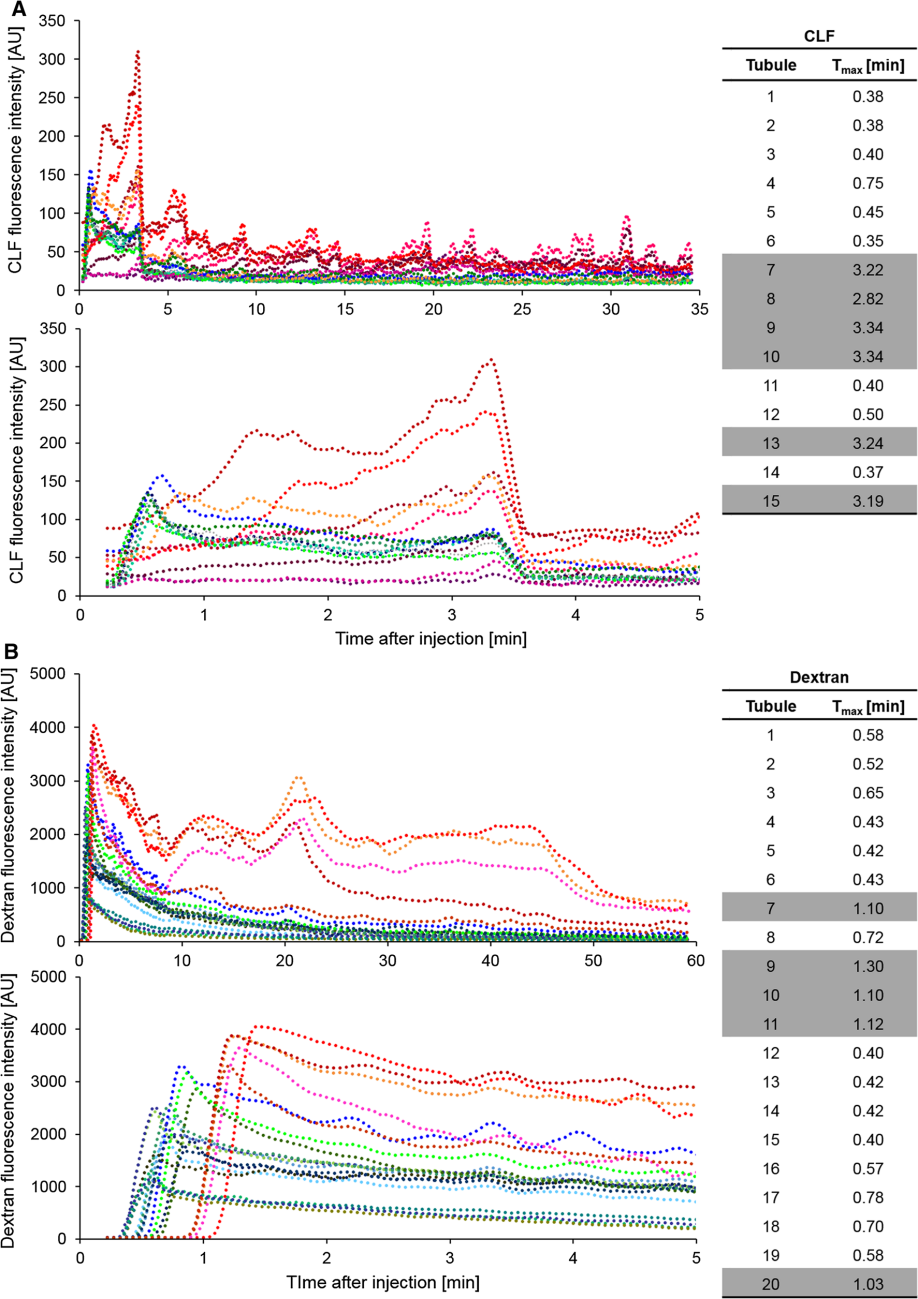
Kinetics of fluorescence differentiate between proximal and distal tubules. Plots representing quantification of CLF (**a**) and dextran (**b**) fluorescence in renal tubules over the total time of imaging (*upper panel*) and the initial phase after administration (*lower panel*). *Each curve* reflects the presence of the fluorescent compound in an individual tubule (*blue* and *green colored lines* represent proximal tubules, *reddish lines* are distal tubules). The calculated time to maximum (*T*_max_, *right side*) separates proximal (*white*) from distal (*gray*) tubules (color figure online)

In order to study the specific transport kinetics of the proximal and distal tubules, we used TMRE to visualize nephron morphology. CLF or fluorescently labeled dextran was injected and imaged (Videos 9, 10). The fluorescent signals of the tubular lumina were then quantified over time (Fig. 7a, b). The calculated time to maximum (*T*_max_) fluorescence in all tubules clearly discriminated a slow and a fast population, corresponding to the proximal and distal tubules, respectively (Fig. 7a, b lower panels). The time to maximum for CLF ranged between 0.38–0.75 min for the proximal and 2.82–3.34 min for the distal tubules (Fig. 7a). For dextran, the discrimination was less distinct with 0.40–0.78 min for the proximal and 1.03–1.30 min for the distal tubules (Fig. 7b). The difference between the two tubule populations reflects the time of passage through the loop of Henle. The differences within the subpopulations can be explained by the variable distance from the glomeruli to the imaged tubule. The distal tubules showed a prolonged and intense signal due to the concentration of the primary urine in the prior Loop of Henle. The kinetics in the distal tubules revealed periodic waves, especially after CLF injection (Fig. 7a, b), which may result from the integrated effect of reabsorption, influx from the proximal tubule as well as efflux to the collecting ducts.

### Intestine

**Fig. 8:**
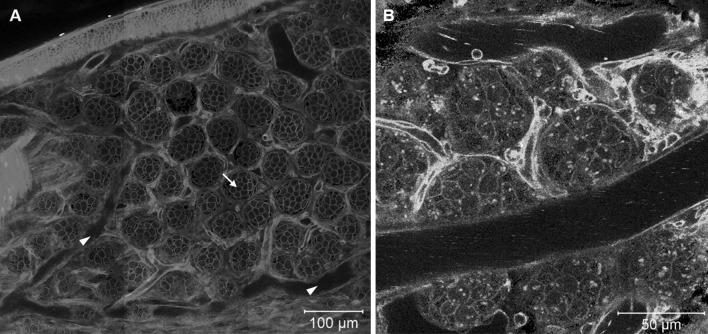
Intravital imaging of the duodenum. **a** Smooth muscle cells surrounding the duodenum, crypts (*arrow*), arterioles/venules (*arrowheads*), and intervillar capillaries are visible in the duodenum of mT/mG mice. **b** In addition, epithelial cells of the blood capillaries, platelets and immune cells are distinguishable in Tie2 reporter mice. *Images* were taken at the level of the crypts as illustrated by the dashed line in Fig. 2c

When imaging the intestine of mT/mG mice, layers of smooth muscle cells, intestinal crypt cells, neighboring capillaries and lymphatic vessels could be distinguished (Fig. 8a). In Tie2 × mT/mG reporter mice, the endothelial cells of the intestinal blood capillaries were also detectable (Fig. 8b). Markers of mitochondrial activity showed strong fluorescence in intestinal epithelial cells and could be used to visualize the morphology of small intestinal crypts (Video 12).

**Fig. 9:**
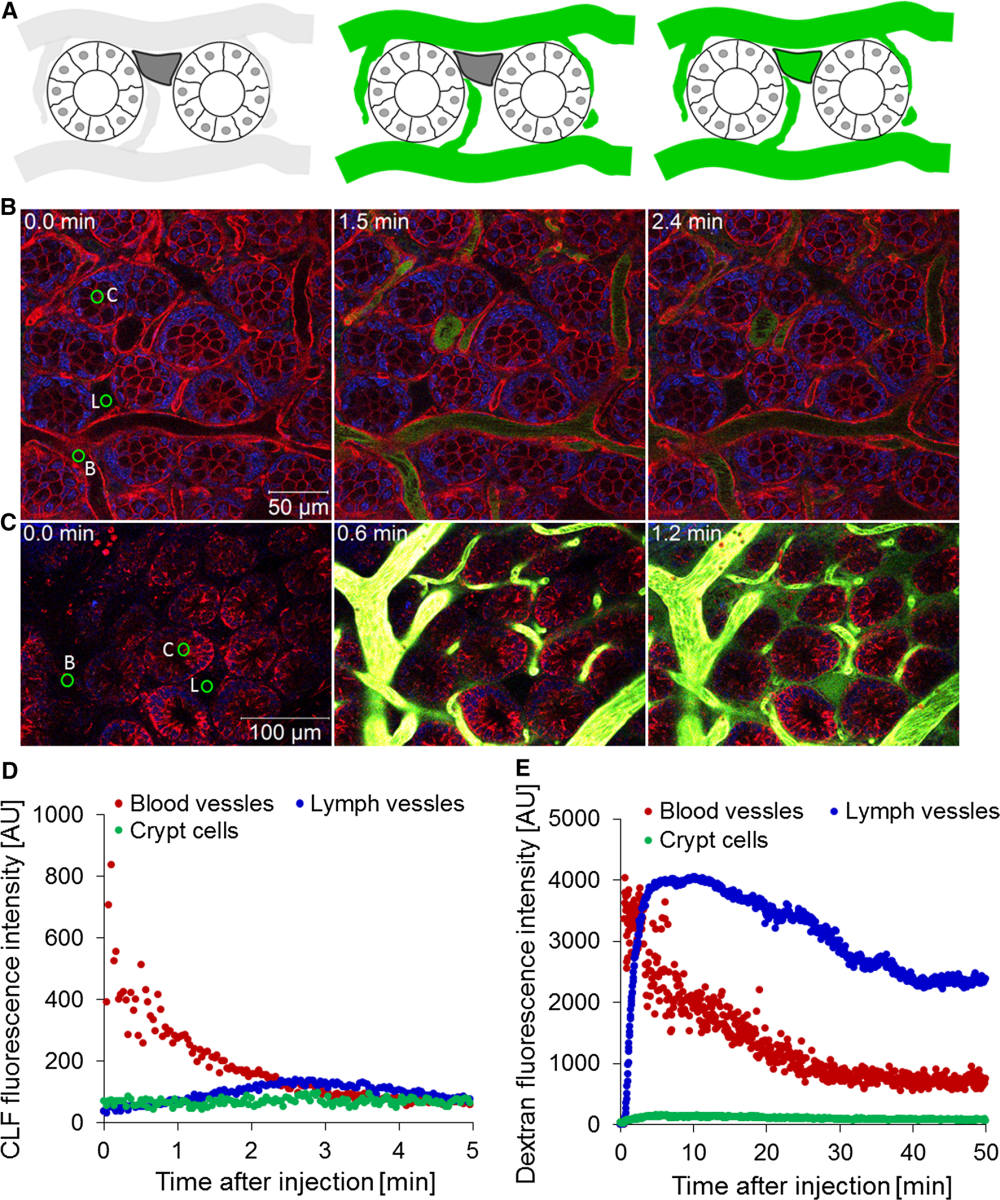
Intestinal transport of CLF and dextran. **a** Illustration of two stages of intestinal transport: appearance in the blood vessels and capillaries and transport into the lacteal. **b** Stills representing these stages after intravenous CLF injection taken from Video 11. Intestinal morphology is visible due to the expression of membrane-targeted tomato (*red*). Transport of CLF was analyzed based on *green* fluorescence. **c** Stills representing the stages after fluorescent dextran injection from Video 12. Intestinal morphology was visualized by TMRE (*red*), and transport of dextran was tracked (*green*). Quantification of CLF (**d**) and dextran (**e**) kinetics in the blood vessels (*B*), lymph vessel/lacteal (*L*) and cryptic cells (*C*). Positions of quantification are indicated by *green* circles in the *left panels* of **b** and **c** (color figure online)

The half-lives of both CLF and dextran in the blood vessels were similar to that in the liver and kidney (Table 2). When CLF was injected, the fluorophore entered the blood capillaries and was not taken up by the crypt cells, but was found in traces in the lymphatic vessels (Fig. 9a, b; Video 11). In contrast, intravenously injected dextran was efficiently taken up into lymphatic vessels as soon as the compound entered the capillaries (Fig. 9c). No transport of CLF or dextran from intestinal capillaries into intestinal crypt cells and no secretion into the intestinal lumen were seen (Fig. 9d, e). This is in contrast to the liver, where hepatocytes have a principally similar polarized structure with the basolateral membrane facing a capillary (the liver sinusoid) and an apical cell pole forming a lumen (in case of hepatocytes the bile canaliculus). Both compounds, CLF and dextran, were efficiently transported along the basolateral-apical axis of hepatocytes and secreted into the lumen. This did not occur in intestinal epithelial cells, because this cell type usually shows an apical to basolateral orientation of transport processes.

Recently, intestinal lacteals have attracted much attention (Bernier-Latmani et al. 2015; Choe et al. 2015; Nurmi et al. 2015). Lacteals represent specialized lymphatic vessels in the center of each villus. They are essential for absorption of fatty acids and lipid soluble vitamins. Moreover, they play a key role in gut immune responses. Recently, an intravital imaging technique for intestinal lacteals has been published, which allowed, for the first time, analysis of lipid drainage and contractility of intestinal lymphatic vessels (Choe et al. 2015). In contrast to the here established two-photon-based imaging of intact intestine, this study required surgical opening of the intestinal lumen. This is important, because cutting the intestine compromises blood flow in the capillaries. The technique used here allows imaging of compound transport between intestinal capillaries and lacteals. Interestingly, penetration into lacteals showed large differences between the tested compounds. Dextran crossed the blood-lacteal barrier within seconds and was significantly enriched in lacteals (Video 12; Fig. 9e). In contrast, the blood-lacteal barrier was virtually impermeable for the fluorescein-coupled bile acid CLF. This differential permeability of the blood-lacteal barrier may be important for design of drugs aimed at influencing the intestinal immune response.

### Immune cells imaging

**Fig. 10:**
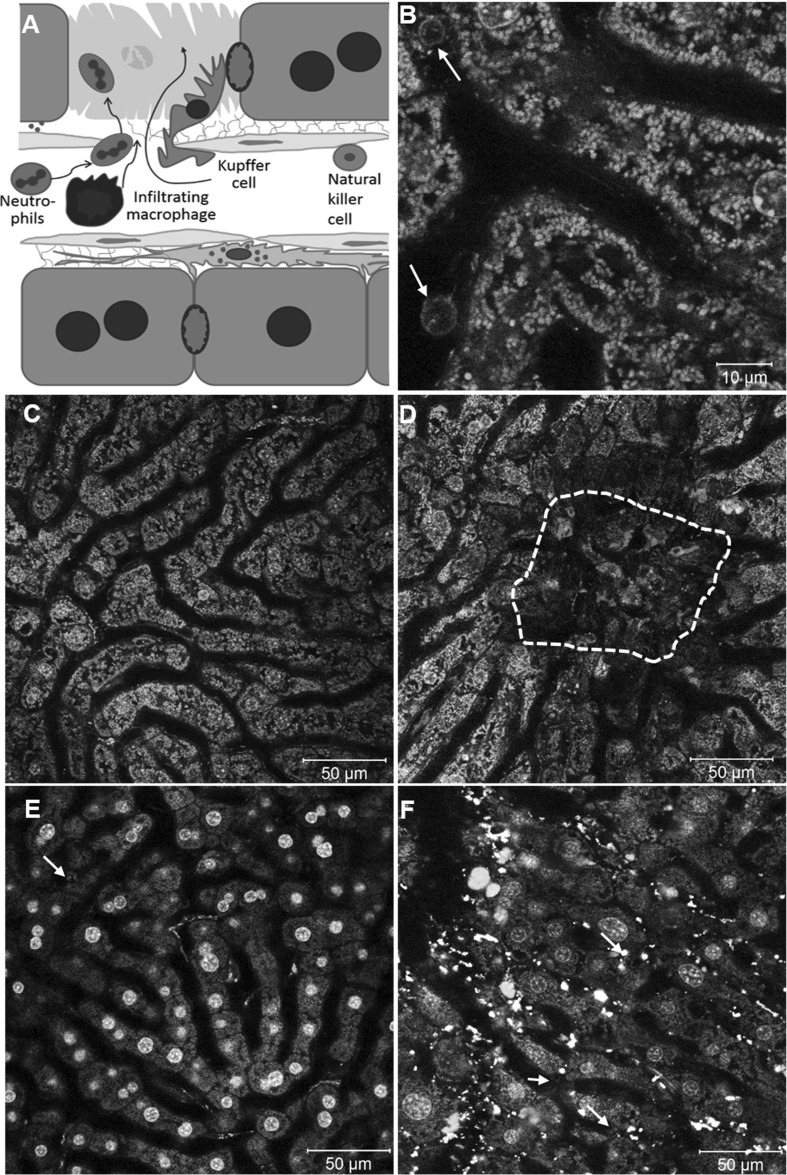
Marking individual immune cell populations with antibodies against specific surface proteins in vivo. **a** Schematic illustration of various immune cell types in the liver infiltrating into a region with dead hepatocytes. **b** Neutrophil staining with a fluorescently labeled Ly6G antibody (*arrows*). Infiltration of macrophages stained by a fluorescently labeled CD11b antibody in a control liver (**c**) and of a liver 3 days after intoxication with acetaminophen (**d**). Infiltration of NK cells stained by fluorescently labeled NK1.1 antibody in a control liver (**e**) and during fibrosis recovery after chronic CCl_4_ administration (**f**)

In addition to resident macrophages such as Kupffer cells in liver or mesangial cells in kidney, circulating immune cells can infiltrate into tissues in large numbers under inflammatory conditions. Usually this causes severe alterations in compound transport, uptake and elimination. For example, hepatic bile acid uptake is decreased during cholestasis (Zollner and Trauner 2008); blood flow and glomerular filtration rate may decrease in acute or chronic nephritis (Imig and Ryan 2013). In order to study compound transport under inflamed conditions, the following section focuses on imaging of neutrophils, infiltrating macrophages and natural killer cells (Fig. 10a) using the same techniques as described above. For this purpose, three types of tissue damage were introduced, physical damage by high laser intensity, as well as acute and chronic tissue damage induced by hepatotoxic compounds.

The LysM × mT/mG reporter mouse represents an appropriate tool to study neutrophil infiltration (Video 4). After induction of physical tissue damage, neutrophils swarm into the damaged region within minutes. While the burned tissue region is densely covered with neutrophils, they also show prolonged residence in the undamaged vicinity (Video 13). Alternatively, neutrophils can be studied by injection of fluorophore-coupled Ly6G antibodies (Fig. 10b).

In order to study infiltration of macrophages during acute liver damage, acetaminophen (APAP) intoxication was applied. These infiltrating myeloid cells (potentially including monocytes, macrophages and dendritic cells) differ from the resident Kupffer cells by the relatively high expression of CD11b (Ramachandran et al. 2012; Zigmond et al. 2014). Intravenous injection of fluorophore-coupled CD11b antibodies into mice 3 days after APAP administration reveals massive infiltration of myeloid cells into the dead cell area compared to control tissue (Fig. 10c, d).

Circulating natural killer (NK) cells can be visualized by intravenous injection of fluorophore-labeled NK1.1 antibodies. In healthy livers, only a relatively small number of NK cells are present in the sinusoids (Fig. 10e). An experimental condition where NK cells play an important role is fibrosis, which can be induced in the liver by chronic administration of carbon tetrachloride (CCl_4_). In the fibrotic liver, NK cells infiltrate into the tissue (Fig. 10f) in order to induce apoptosis of myofibroblasts during fibrosis recovery (Fasbender et al. 2016).

It should be considered that antibody-based visualization of immune cells might alter functionality of the labeled cells or trigger further immune cell responses by the bound antibody; therefore, this approach might best be limited to short-term imaging. In contrast, recording based on reporter mice should be the first choice when prolonged imaging is required.

### Future perspectives

The presented two-photon-based imaging setup describes intravital imaging of the most important organs involved in uptake and elimination of endogenous molecules as well as xenobiotics. Before using fluorescently labeled compounds in such studies, it must be ensured that the modified compound behaves similar to the original, as it was reported for CLF (de Waart et al. 2010).

Under optimal conditions image resolution sufficient for tracing individual vesicles, cell organelles such as mitochondria or even endocytic processes can be achieved. This opens possibilities to gain a deeper understanding of transport processes. For example, in the presented data CLF transport from the liver sinusoids to bile canaliculi includes a transient enrichment in a narrow space close to the basolateral hepatocyte membrane, which was not detected before. Also analysis of the transport from the intestinal capillaries to lacteals has not been possible in uncut intestine in the past, due to limitations of laser penetration and scattering of light. Two-photon imaging has been applied previously to study glomerular filtration (Nakano et al. 2012; Sandoval and Molitoris 2013) and transport in renal tubules (Wagner et al. 2016). The new technique described here allows simultaneous imaging of glomerular capillaries, Bowman’s space and proximal and distal tubules. This offers the opportunity to model excretion by the nephron as a function of secretion, reuptake and flow in each of these compartments simultaneously.

The presented studies of compound transport in tissues can, in particular, support physiologically based computational models. Currently, first-in-human trials represent a critical step in drug development, because of interspecies differences and difficulties of extrapolation from mouse to human (Thiel et al. 2015). Recently, PBPK modeling for cross-species extrapolation has been introduced, which improved the accuracy of prediction by incorporating target species-specific model parameter domains, such as species-specific physiology, plasma protein binding, transport kinetics and metabolism (Thiel et al. 2015). A limitation is that current PBPK models usually consider individual organs only as single compartments. However, the present study shows that transport occurs in various sub-compartments of tissues, which can be independently quantified. The measurements provide an important systems view on drug distribution within the organism: On the one hand, the blood concentrations in the different organs reflect drug concentration profiles in the overall blood pool of the body. On the other hand, the profiles in the specific sub-compartments enable a quantitative assessment of drug distribution within specific tissue compartments. Such measurements allow for the targeted quantification of specific physiological processes in detailed computational models of the liver (Ghallab et al. 2016; Hoehme et al. 2010; Schwen et al. 2014; Vartak et al. 2016), the kidney (Thomas 2009) or the intestine (Thelen et al. 2011). Another interesting application could be validation and refinement of distribution models describing organ-plasma partitioning in PBPK modeling (Jones et al. 2009). In this regard, it should be noted that the given profiles may be used for the derivation of biliary and renal clearance rates, as such enabling the quantitative assessment of drug excretion. These measurements therefore provide important information to key physiological processes underlying drug pharmacokinetics, i.e., distribution and excretion.

Many compounds such as bile acids, drugs and toxins undergo enterohepatic circulation and may be modified by the microbiota of the intestine or by the metabolism of the hepatocytes (Ridlon et al. 2015; Roberts et al. 2002). This kind of modification can alter pharmacological activities and transport efficiency. Measuring compound uptake in the intestine as well as in the hepatocytes in vivo as described in this study can give information on intestinal impermeability, hepatic uptake and body clearance of the parent substance and its metabolite, which may be dependent upon the properties of the liver-gut axis.

An attractive future application of the introduced systemic imaging method is to integrate spatial–temporal fluorescence image correlation methods in vivo such as fluorescence correlation spectroscopy (FCS) (Elson 2001) and raster image correlation spectroscopy (RICS) (Brown et al. 2008). The recording setup fulfills many important preconditions for FCS and RICS such as stable focus, fast imaging sequences and low phototoxicity. These techniques allow the determination of absolute concentrations of fluorescent test compounds in the analyzed compartments. Besides compound concentrations, correlation spectroscopy can yield further interesting data on protein binding, flux, diffusion coefficients and orientation of transport (Brown et al. 2008; Rossow et al. 2010).

In conclusion, the two-photon-based imaging toolbox presented here allows quantitative spatial–temporal analysis of physiological as well as pathophysiological processes in intact liver, kidney and intestine.

## Electronic supplementary material

Below is the link to the electronic supplementary material.

Supplementary material 1 (AVI 11163 kb)

Supplementary material 2 (AVI 8408 kb)

Supplementary material 3 (AVI 5947 kb)

Supplementary material 4 (AVI 102963 kb)

Supplementary material 5 (AVI 19240 kb)

Supplementary material 6 (AVI 249090 kb)

Supplementary material 7 (AVI 59719 kb)

Supplementary material 8 (AVI 46695 kb)

Supplementary material 9 (MP4 28056 kb)

Supplementary material 10 (MP4 148118 kb)

Supplementary material 11 (MP4 102689 kb)

Supplementary material 12 (MP4 59130 kb)

Supplementary material 13 (AVI 65085 kb)

Supplementary material 14 (MP4 65901 kb)

Supplementary material 15 (MP4 26229 kb)

Supplementary material 16 (DOCX 12 kb)

## Abbreviations

TMRE: Tetramethylrhodamine ethylester
PBPK: Physiologically based pharmacokinetic
PP: Periportal
PC: Pericentral
LSEC: Liver sinusoidal endothelial cell
CLF: Cholyl-lysyl-fluorescein

## References

[CR1] Beattie L, d’El-Rei Hermida M, Moore JW (2013). A transcriptomic network identified in uninfected macrophages responding to inflammation controls intracellular pathogen survival. Cell Host Microbe.

[CR2] Bernier-Latmani J, Cisarovsky C, Demir CS (2015). DLL4 promotes continuous adult intestinal lacteal regeneration and dietary fat transport. J Clin Invest.

[CR3] Brown CM, Dalal RB, Hebert B, Digman MA, Horwitz AR, Gratton E (2008). Raster image correlation spectroscopy (RICS) for measuring fast protein dynamics and concentrations with a commercial laser scanning confocal microscope. J Microsc.

[CR4] Choe K, Jang JY, Park I (2015). Intravital imaging of intestinal lacteals unveils lipid drainage through contractility. J Clin Invest.

[CR5] Clausen BE, Burkhardt C, Reith W, Renkawitz R, Forster I (1999). Conditional gene targeting in macrophages and granulocytes using LysMcre mice. Transgenic Res.

[CR6] de Waart DR, Hausler S, Vlaming ML (2010). Hepatic transport mechanisms of cholyl-l-lysyl-fluorescein. J Pharmacol Exp Ther.

[CR7] Denk W, Strickler JH, Webb WW (1990). Two-photon laser scanning fluorescence microscopy. Science.

[CR8] Elson EL (2001). Fluorescence correlation spectroscopy measures molecular transport in cells. Traffic.

[CR9] Fasbender F, Widera A, Hengstler JG, Watzl C (2016). Natural killer cells and liver fibrosis. Front Immunol.

[CR10] Ghallab A, Celliere G, Henkel SG (2016). Model-guided identification of a therapeutic strategy to reduce hyperammonemia in liver diseases. J Hepatol.

[CR11] Helmchen F, Denk W (2005). Deep tissue two-photon microscopy. Nat Methods.

[CR12] Hoehme S, Brulport M, Bauer A (2010). Prediction and validation of cell alignment along microvessels as order principle to restore tissue architecture in liver regeneration. Proc Natl Acad Sci USA.

[CR13] Imig JD, Ryan MJ (2013). Immune and inflammatory role in renal disease. Compr Physiol.

[CR14] Inverso D, Iannacone M (2016). Spatiotemporal dynamics of effector CD8+ T cell responses within the liver. J Leukoc Biol.

[CR15] Jones HM, Gardner IB, Watson KJ (2009). Modelling and PBPK simulation in drug discovery. AAPS J.

[CR16] Koni PA, Joshi SK, Temann UA, Olson D, Burkly L, Flavell RA (2001). Conditional vascular cell adhesion molecule 1 deletion in mice: impaired lymphocyte migration to bone marrow. J Exp Med.

[CR17] Lammermann T, Afonso PV, Angermann BR (2013). Neutrophil swarms require LTB4 and integrins at sites of cell death in vivo. Nature.

[CR18] Marques PE, Oliveira AG, Pereira RV (2015). Hepatic DNA deposition drives drug-induced liver injury and inflammation in mice. Hepatology.

[CR19] Marques PE, Antunes MM, David BA, Pereira RV, Teixeira MM, Menezes GB (2015). Imaging liver biology in vivo using conventional confocal microscopy. Nat Protoc.

[CR20] Masedunskas A, Weigert R (2008). Intravital two-photon microscopy for studying the uptake and trafficking of fluorescently conjugated molecules in live rodents. Traffic.

[CR21] Melgar-Lesmes P, Edelman ER (2015). Monocyte-endothelial cell interactions in the regulation of vascular sprouting and liver regeneration in mouse. J Hepatol.

[CR22] Milkiewicz P, Saksena S, Cardenas T, Mills CO, Elias E (2000). Plasma elimination of cholyl-lysyl-fluorescein (CLF): a pilot study in patients with liver cirrhosis. Liver.

[CR23] Muzumdar MD, Tasic B, Miyamichi K, Li L, Luo L (2007). A global double-fluorescent Cre reporter mouse. Genesis.

[CR24] Nakano D, Kobori H, Burford JL (2012). Multiphoton imaging of the glomerular permeability of angiotensinogen. J Am Soc Nephrol.

[CR25] Nurmi H, Saharinen P, Zarkada G, Zheng W, Robciuc MR, Alitalo K (2015). VEGF-C is required for intestinal lymphatic vessel maintenance and lipid absorption. EMBO Mol Med.

[CR26] Pittet MJ, Weissleder R (2011). Intravital imaging. Cell.

[CR27] Pupyshev AB, Maiborodina VI (2002). Sucrose-stimulated release of FITC-dextran into the bile in the dynamics of its storage and elimination from the liver. Bull Exp Biol Med.

[CR28] Ramachandran P, Pellicoro A, Vernon MA (2012). Differential Ly-6C expression identifies the recruited macrophage phenotype, which orchestrates the regression of murine liver fibrosis. Proc Natl Acad Sci USA.

[CR29] Ridlon JM, Kang DJ, Hylemon PB, Bajaj JS (2015). Gut microbiota, cirrhosis, and alcohol regulate bile acid metabolism in the gut. Dig Dis.

[CR30] Roberts MS, Magnusson BM, Burczynski FJ, Weiss M (2002). Enterohepatic circulation: physiological, pharmacokinetic and clinical implications. Clin Pharmacokinet.

[CR31] Rossow MJ, Sasaki JM, Digman MA, Gratton E (2010). Raster image correlation spectroscopy in live cells. Nat Protoc.

[CR32] Sandoval RM, Molitoris BA (2013). Quantifying glomerular permeability of fluorescent macromolecules using 2-photon microscopy in Munich Wistar rats. J Vis Exp.

[CR33] Schwen LO, Krauss M, Niederalt C (2014). Spatio-temporal simulation of first pass drug perfusion in the liver. PLoS Comput Biol.

[CR34] Smith AM, Mancini MC, Nie S (2009). Bioimaging: second window for in vivo imaging. Nat Nanotechnol.

[CR35] Thelen K, Coboeken K, Willmann S, Burghaus R, Dressman JB, Lippert J (2011). Evolution of a detailed physiological model to simulate the gastrointestinal transit and absorption process in humans, part 1: oral solutions. J Pharm Sci.

[CR36] Thiel C, Schneckener S, Krauss M (2015). A systematic evaluation of the use of physiologically based pharmacokinetic modeling for cross-species extrapolation. J Pharm Sci.

[CR37] Thomas SR (2009). Kidney modeling and systems physiology. Wiley Interdiscip Rev Syst Biol Med.

[CR38] Vartak N, Damle-Vartak A, Richter B (2016). Cholestasis-induced adaptive remodeling of interlobular bile ducts. Hepatology.

[CR39] Wagner MC, Campos-Bilderback SB, Chowdhury M (2016). Proximal tubules have the capacity to regulate uptake of albumin. J Am Soc Nephrol.

[CR40] Wolf K, Mazo I, Leung H (2003). Compensation mechanism in tumor cell migration: mesenchymal-amoeboid transition after blocking of pericellular proteolysis. J Cell Biol.

[CR41] Zigmond E, Samia-Grinberg S, Pasmanik-Chor M (2014). Infiltrating monocyte-derived macrophages and resident kupffer cells display different ontogeny and functions in acute liver injury. J Immunol.

[CR42] Zipfel WR, Williams RM, Webb WW (2003). Nonlinear magic: multiphoton microscopy in the biosciences. Nat Biotechnol.

[CR43] Zollner G, Trauner M (2008). Mechanisms of cholestasis. Clin Liver Dis.

