# Supplementary material for: In vivo imaging of systemic transport and elimination of xenobiotics and endogenous molecules in mice
Source: Arch Toxicol. 2016 Dec 20;91(3):1335–52. doi: 10.1007/s00204-016-1906-5 (PMC5316407; doi:10.1007/s00204-016-1906-5)
Supplement: Supplementary file 16 — Supplementary material 16 (DOCX 12 kb) [file 204_2016_1906_MOESM16_ESM.docx]

**Video legends**

**Video 1: Visualization of trafficking vesicles by mT/mG mice.** (**A)** Imaging of the liver at 800 nm visualizes vesicles which cluster close to the apical hepatocyte membrane. **(B)** Higher magnification allows tracking of individual trafficking vesicles.

**Video 2: Liver morphology by administration of a mitochondrial membrane potential marker.** Mitochondria of hepatocytes and a Kupffer cell (arrow) in a wildtype mouse following uptake of Rhodamine 123.

**Video 3:** **LSEC in a Tie2 x mT/mG reporter mouse.** The arrow indicates the nucleus of a LSEC. Moreover, platelets and some immune cells also express eGFP.

**Video 4:** **Kupffer cells expressing eGFP in a LysM x mT/mG reporter mouse.**

**Video 5: Hepatic transport of CLF.** Wildtype mouse pre-injected with TMRE (red) was imaged after tail vein injection of CLF (2.5 mg/kg).

**Video 6: Hepatic transport of dextran.** Wildtype mouse pre-injected with fluorescently labeled wheat germ agglutinin (red) was imaged following the tail vein injection of fluorescently labeled dextran (30 mg/kg).

**Video 7: Glomerular filtration of CLF and tubular transport.** A mT/mG mouse (red) was imaged after tail vein injection of CLF.

**Video 8: Glomerular filtration of dextran and tubular transport.** A mT/mG mouse (red) was imaged after tail vein injection of fluorescently labeled dextran (30 mg/kg).

**Video 9: Renal transport of CLF.** Wildtype mouse pre-injected with TMRE (red) was imaged after tail vein injection of CLF (2.5 mg/kg).

**Video 10: Renal transport of dextran.** Wildtype mouse pre-injected with TMRE (red) was imaged after tail vein injection of fluorescently labeled dextran (30 mg/kg).

**Video 11: Intestinal transport of CLF.** mT/mG mouse (red) was imaged after tail vein injection of CLF (2.5 mg/kg).

**Video 12: Intestinal transport of fluorescently labeled dextran.** Wildtype mouse pre-injected with TMRE (red) was imaged after tail vein injection of fluorescently labeled dextran (30 mg/kg).

**Video 13: Neutrophils swarming after physical liver damage.** A LysM x mT/mG mouse was treated with high energy laser at the indicated region (circle) 6 minutes into the imaging window and infiltrating neutrophils (green) were imaged.
